# Morphometric Evaluation of Sutural Patterns at the Pterion and Asterion in Dry Indian Skulls: Surgical Relevance

**DOI:** 10.7759/cureus.54466

**Published:** 2024-02-19

**Authors:** Rahul Sharma, Vikas Vaibhav, Raviprakash Meshram, Gitanjali Khorwal, Brijendra Singh, Yashu Bhardwaj

**Affiliations:** 1 Anatomy, All India Institute of Medical Sciences, Rishikesh, IND; 2 Forensic Medicine and Toxicology, All India Institute of Medical Sciences, Rishikesh, IND; 3 Anatomy, All India Institute of Medical Sciences, Kalyani, IND

**Keywords:** morphometric evaluation, anthropometric study, pterion & asterion surgical perspective, morphometric analysis of asterion, morphometric analysis of pterion

## Abstract

Introduction: The pterion and asterion serve as crucial landmarks on the skull, representing the antero-lateral and postero-lateral fontanelles in neonates, respectively. In clinical practice, these points play a pivotal role in guiding the understanding of deeper structures and their relationships to the head's surface. The thin calvarium at these junctures is susceptible to fractures, and the underlying vessels are prone to tear, often leading to extradural hematoma formation, necessitating burr hole surgery for evacuation.

Material and methods: The study involved 40 human dry skulls in Indians (n= 27 male (10.8%), n= 13 female (5.2%)) of unknown age, evaluating morphometric characteristics of 80 pterions and asterions. Measurements were conducted using a digital caliper (SKADIOO±0.2mm/0.01") in millimeters on both sides of each skull. The investigation also included an examination of sutural patterns in Pterion and asterion.

Results: Three types of sutural patterns were identified in the pterion, the most common being the sphenoparietal variety (75% on the right, 70% on the left), followed by the epipteric variety, which was the second most common (11.3% on the right, 12.5% on the left), and then the frontotemporal type (1.3% on the right, 2.5% on the left). Two sutural patterns were observed in the asterion: type 1 (presence of sutural bone) in 17.9% of skulls and type 2 (absence of sutural bone) in 82.1%.

Conclusion: The differences in pterion and asterion positions across various populations explored in previous studies motivated us to conduct this research in the Indian population. Our findings revealed that among Indians, the predominant pterion type is predominantly sphenoparietal, whereas type 2 is the most prevalent in asterion. Understanding the clinical significance of the pterion and asterion is crucial for healthcare professionals to ensure precise and safe surgical procedures, particularly for the effective treatment of head trauma patients.

## Introduction

The pterion, a significant anatomical landmark, is formed by the convergence of the frontal bone, parietal bone, squama temporalis, and the greater wing of the sphenoid bone, shaping the floor of the temporal fossa. In neonatal skulls, it corresponds to the anterolateral fontanelle, typically closing by the third month after birth [1]. This area primarily serves as a reference point for explaining the positioning of deeper structures and their relationships to the surface of the head [2]. Its clinical significance stems from the thin and easily fractured nature of the calvarium situated above the anterior branch of the middle meningeal artery, making it prone to damage and resulting in extradural hematoma, often necessitating surgical intervention such as burr hole surgery [2].

Other structures associated with the pterion include the middle cerebral artery, the anterior pole of the insula, and Broca's area. Additionally, an oblique line drawn from the fronto-zygomatic suture to the pterion delineates the inferior border of the frontal lobe [3].

The asterion marks the junction of the lambdoid, parietomastoid, and occipitomastoid sutures, where the posterolateral fontanelle is located at birth, typically closing within the first six to eight months of life [4]. It also lies just above the Frankfurt horizontal plane [5]. It is intricately linked to the confluence of the transverse and sigmoid sinuses and serves as a crucial reference point for a lateral approach to the posterior cranial fossa in surgical procedures [6,7]. Surgical intervention is often necessary for various concerns in the posterior cranial fossa, including vascular, inflammatory, neoplastic, or traumatic lesions [5,7]. Understanding the surface protrusion of deep anatomical structures is essential for surgeons working with these structures, with the pterion and asterion serving as reliable surface points for revealing fundamental structures [8].

Locating the pterion and asterion during emergency evacuation procedures can be challenging due to their concealment beneath the scalp [1]. Therefore, this study aimed to determine their positions using external landmarks.

## Materials and methods

This descriptive anthropometric study was conducted at the Department of Anatomy, All India Institute of Medical Science and Research, Rishikesh, Uttarakhand. The research involved 40 human dry skulls, comprising 27 male (10.8%) and 13 female (5.2%) skulls of unknown age, all devoid of bony malformations or signs of trauma scars. Morphometric characteristics of 80 pterions and asterions were assessed on both sides of the 40 skulls.

**Figure 1:**
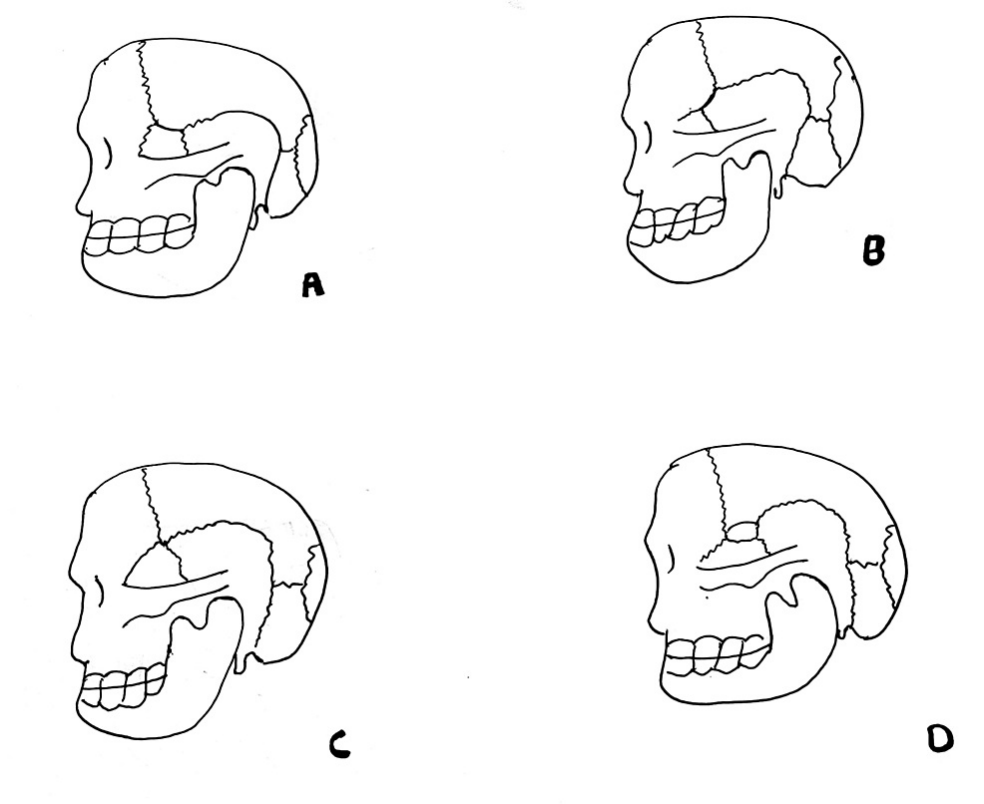
Type of sutural pattern in the pterion A- Sphenoparietal type (sphenoid and parietal are in direct contact), B- Frontotemporal type (frontal and temporal bone are in direct contact), C- Stellate type ( frontal, parietal, temporal & sphenoidal are in direct contact), D- Epipteric type (presence of small sutural bone between parietal bone & greater wing of sphenoid) Image credit: Rahul Sharma

The investigation focused on determining each skull's sutural patterns of the pterion and asterion. Following Murphy's classification, the sphenoparietal type was identified as a sutural pattern where the sphenoid and parietal bones are in direct contact (Figure 1A), frontotemporal type represents a sutural pattern where the frontal and temporal bones are in direct contact (Figure 1B), stellate type featured the articulation of four bones (frontal, parietal, temporal, and sphenoid) at a single mark (Figure 1C) and The epipteric type was characterized by a small sutural bone between the parietal bone and the greater wing of the sphenoid bone (Figure 1D) [9].

**Figure 2:**
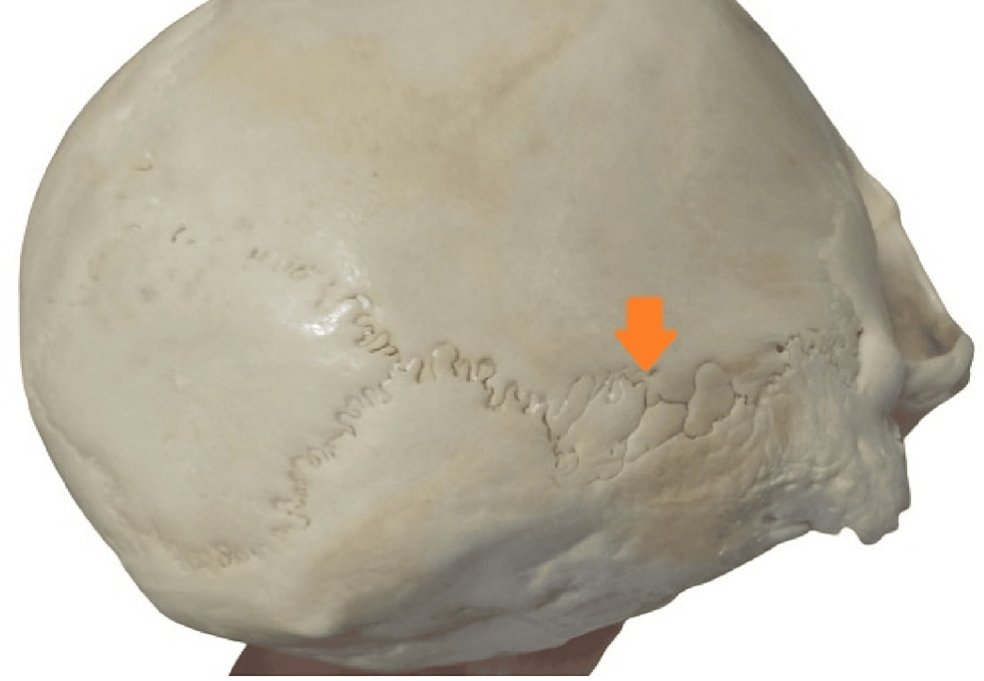
T1 (presence of sutural bone at the asterion)

**Figure 3:**
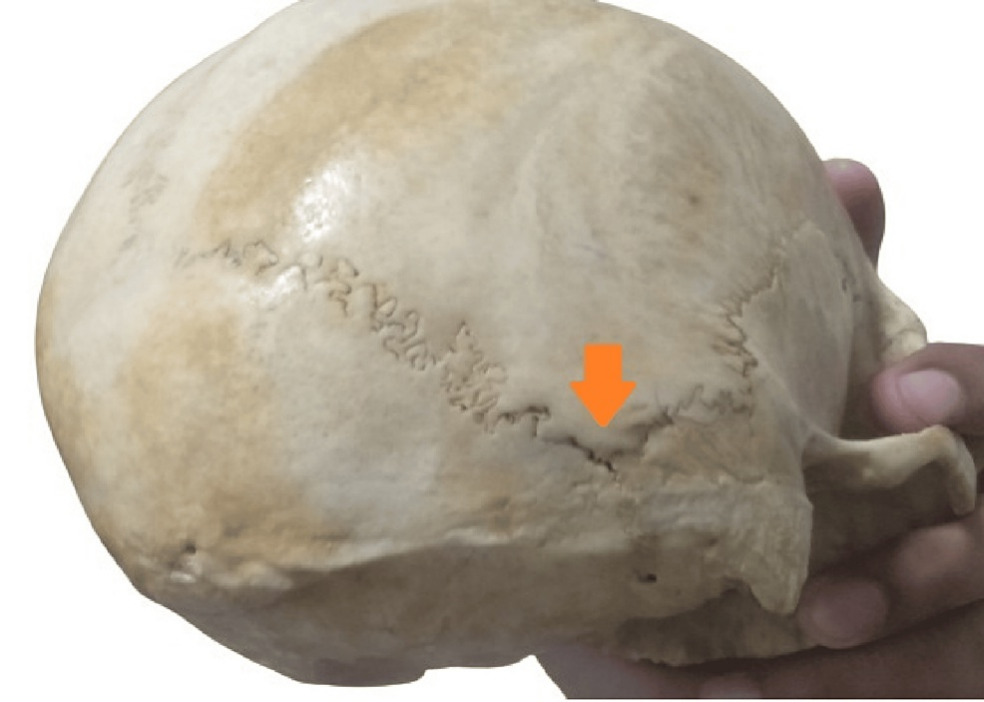
T2 (absence of sutural bone at the asterion)

There are two categories for the sutural morphology of the asterion: T1 (sutural bone present at the asterion) and T2 (sutural bone absent at the asterion) (Figures 2-3).

In this research, a morphological evaluation of various cranial and mandibular features, including the supraorbital ridge, supraorbital margin, orbital form, zygomatic arc, frontal eminence, mastoid process, inferior nuchal line, mental eminence, and masseteric tuberosity, was employed to determine the physical differences between the two biological sexes to determine the sex of human skeletal remains [8].

Measurements were conducted using a digital caliper, recorded in millimeters on both sides of the skull. To mitigate inter-observer errors, a single researcher performed all measurements.

**Figure 4:**
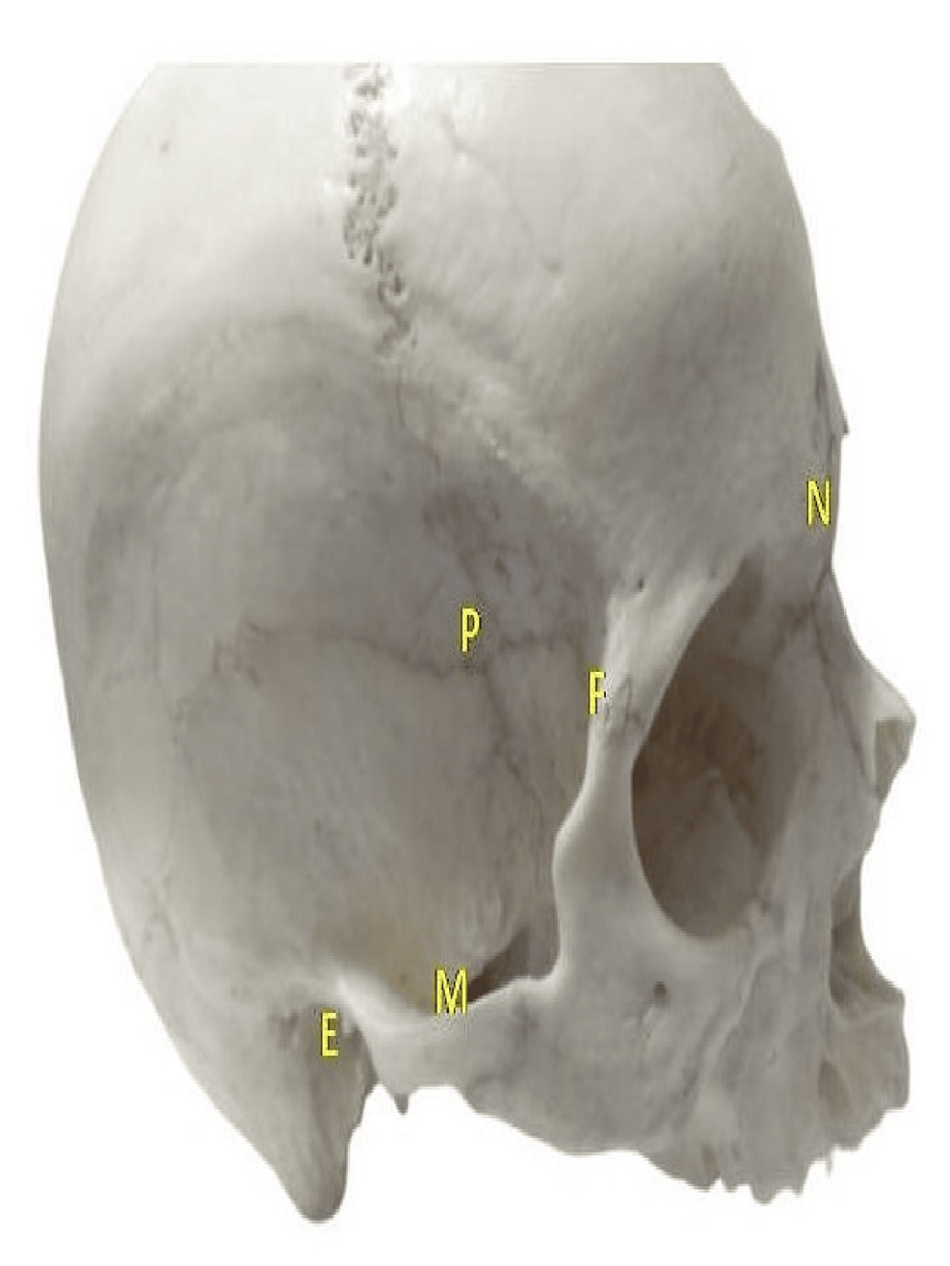
Measurement of the pterion (P) to the midpoint of the zygomatic arch (M); to the posterior aspect of the frontozygomatic suture (F); to the external acoustic meatus (E) and nasion (N)

For pterion measurements, distances were recorded from the pterion (P) to the midpoint of the zygomatic arch (M), to the posterior aspect of the frontozygomatic suture (F), to the external acoustic meatus (E), and the nasion (N) as illustrated in Figure 4.

**Figure 5:**
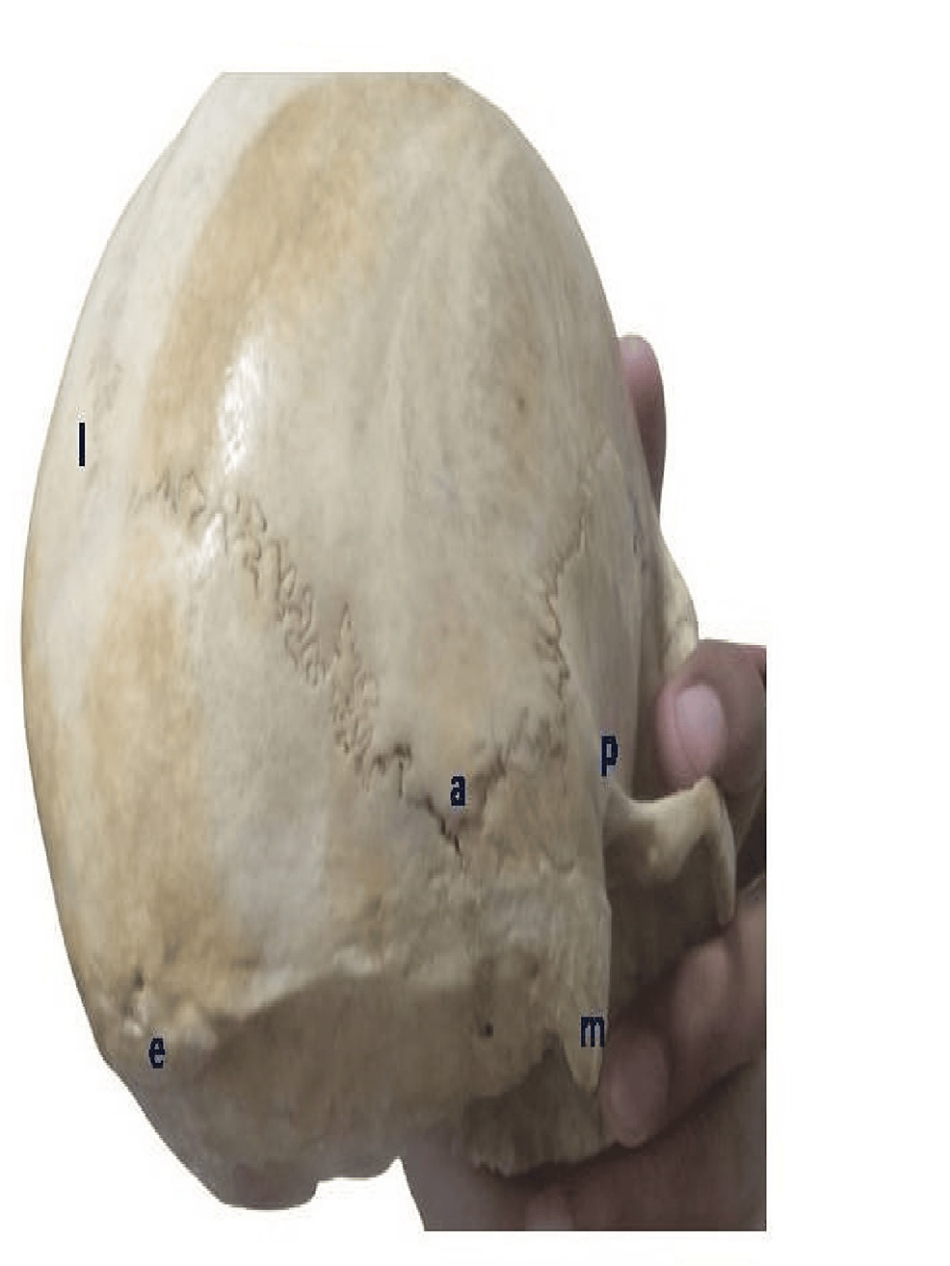
Measurement for the asterion (a) was taken from the asterion to the apex of the mastoid process (m); to the posterior end of the zygomatic arch (p), to the external occipital protuberance (e), and to the lambda (l)

Measurements for the asterion (a) were taken from the asterion to the apex of the mastoid process (m), to the posterior end of the zygomatic arch (p), to the external occipital protuberance (e), and the lambda (l) as shown in Figure 5.

The collected data were organized and tabulated in a Microsoft Excel spreadsheet (Microsoft Corporation, Redmond, WA, USA) and subsequent statistical analyses were performed using descriptive statistics, frequency distribution, and a chi-square contingency table. The Statistical Package for the Social Sciences (SPSS) version 22 (IBM Corp., Armonk, NY, USA) facilitated these analyses. A significance level of p<0.05 was deemed statistically significant.

## Results

The present study observed three types of sutural patterns for the pterion. The most common was the sphenoparietal variety (Figure 1A), with a frequency of 30 (75%) on the right side and 28 (70%) on the left side. The second most common variety was epipteric (Figure 1C), with a frequency of 9 (11.3%) on the right side and 10 (12.5%) on the left side of the skull. The third common was the frontotemporal type, with a frequency of one (1.3%) on the right and two (2.5%) on the left. No skull exhibited the stellate type of the pterion in the study. The frequencies of the different types of the pterion on both the right and left sides of the skull are given in Table 1.

**Table 1:** Frequency distribution of the type of pterion observed on both sides of the skull

Side	Sphenoparietal type	Frontotemporal type	Epipteric type	Stellate type
Right	30 (75%)	1 (1.3%)	9 (11.3%)	0
Left	28 (70%)	2 (2.5%)	10 (12.5%)	0
Total	58 (72.5%)	3 (3.8%)	19 (23.8%)	0

Table 2 presents the findings of the association test that used a chi-square contingency table to look at the relationship between the pterion type and side. The degree of correlation between the pterion type and the side of the skull, as shown by the chi-square value, did not reach statistical significance.

**Table 2:** Chi-square contingency table between the pterion type and side

Side	Sphenoparietal	Frontotemporal	Epipteric	Stellate	X2	df	p-value
Right	30 (75%)	1 (1.3%)	9 (11.3%)	0	0.634	1	0.669
Left	28 (70%)	2 (2.5%)	10 (12.5%)	0			

Table 3 provides descriptive statistics for the distance between the pterion (P) and the midpoint of the zygomatic arch (MZA), to the posterior aspect of the frontozygomatic suture (FZS), to the external acoustic meatus (EA) and nasion (N). The average distance between the pterion and midpoint of the zygomatic arch was 37.33±4.8 mm taking the two sides together, while the average distance on the right side was 37.69±5.01 mm and 36.96±4.8 mm on the left side of the skull. The average distance of the pterion with the posterolateral aspect of the frontozygomatic suture was 29.56±5.9 mm taking the two sides together, while the average distance on the right side was 30.42±5.8 mm and 28.69±5.9 mm on the left side. The mean distance between the pterion from the nasion was 75.29±5.6 mm, taking the two sides together, while the average distance on the right side was 76.04±5.6 mm and 74.54±5.6 mm on the left side. The mean distance of the pterion from the external acoustics meatus was 52.65±3.5 mm taking the two sides together, while the average distance on the right side was 52.82 ±3.2 mm and 52.48± 3.8 mm on the left side of the skull.

**Table 3:** Descriptive statistics of dimensions measured between the pterion and landmarks P: pterion; MZA: midpoint of the zygomatic arch; FZS: frontozygomatic suture; N: nasion; EA: external acoustic meatus

Side	Parameters	n (Sample size)	Minimum (mm)	Maximum (mm)	Mean (mm)	SD
Right	P MZA	40	25.59	47.57	37.69	5.01
	P FZS	40	19.99	45.68	30.42	5.89
	P N	40	66.00	86.64	76.04	5.60
	P EA	40	45.64	60.24	52.82	3.26
Left	PM ZA	40	27.44	46.17	36.96	4.82
	P FZS	40	15.59	37.42	28.69	5.91
	P N	40	60.93	81.56	74.54	5.62
	P EA	40	44.44	59.50	52.48	3.83
Total	P MZA	80	25.59	47.57	37.33	4.88
	P FZS	80	15.59	45.68	29.56	5.91
	P N	80	60.93	86.64	75.29	5.61
	P EA	80	44.44	60.24	52.65	3.52

Both types of sutural patterns were observed in which a total of 7 (17.9%) of the skull were type 1 (presence of sutural bone at the asterion) and 33 (82.1%) were type 2 (absence of sutural bone at the asterion) as shown in Figures 2A, 2B.

Table 4 presents the descriptive statistics of the distance between the asterion (A) and the apex of the mastoid (AOM) to the posterior end of the zygoma (PEZ), external occipital protuberance (EOP), and lambda (L). The mean distance between the asterion and apex of the mastoid was 45.85±5.8 mm taking the two sides together, while the average distance on the right side was 46.41±5.86 mm and 45.30±5.9 mm on the left side of the skull. The mean distance of the asterion with the posterior end of the zygoma was 56.31±5.9 mm taking the two sides together, while the average distance on the right side was 55.72±6.1 mm and 56.90±5.9 mm on the left side. The mean distance between the asterion with external occipital protuberance was 61.08±5.6 mm, taking the two sides together. In contrast, the average distance on the right side was 60.01±5.2 mm and 62.15±5.9 mm on the left side. The mean distance of asterion from lambda was 78.37±4.7 mm, taking the two sides together, while the average distance on the right side was 77.71 ±5.5 mm and 79.03± 3.7 mm on the left side of the skull.

**Table 4:** Descriptive statistics of dimensions measured between the asterion and landmarks A: asterion; AOM: apex of mastoid; PEZ: posterior end of zygoma; EO: external occipital protuberance; L: lambda

Side	Parameters	n (Sample size)	Minimum (mm)	Maximum (mm)	Mean (mm)	SD
Right	A AOM	40	40.50	60.00	46.41	5.86
	A PEZ	40	50.10	70.20	55.72	6.11
	A EO	40	50.70	70.50	60.01	5.23
	A L	40	70.30	90.00	77.71	5.59
Left	A AOM	40	40.20	60.10	45.30	5.99
	A PEZ	40	50.20	70.50	56.90	5.98
	A EO	40	50.70	70.40	62.15	5.97
	A L	40	70.70	83.00	79.03	3.78
Total	A AOM	80	40.20	60.10	45.85	5.86
	A PEZ	80	50.10	70.50	56.31	5.99
	A EO	80	50.70	70.50	61.08	5.64
	A L	80	70.30	90.00	78.37	4.75

## Discussion

In this study, three types of pterions were identified as most prevalent: sphenoparietal, frontotemporal, and epipteric. Among these, the sphenoparietal type was the most dominant, accounting for 72.5% of cases, followed by the epipteric type at (23.8%), and the frontotemporal type was the least common at 3.8%. Regarding asterion types, type 2 (characterized by the absence of sutural bone) was more commonly observed, accounting for (82.1%) of cases, while type 1 (characterized by the presence of sutural bone) was less frequent at (17.9%). Previous research has similarly highlighted the predominance of the sphenoparietal form, with the percentage distribution of the other pterion types varying across different populations, as illustrated in Table 5.

**Table 5:** The 18 studies mentioned

Study	N (Sample size)	Spheno–parietal	Fronto–temporal	Stellate	Epipteric
Australian Aborigines Murphy- 1956 [9]	388	73%	7.50%	18.50%	1%
Nigerian - Saxena et al. 1988 [10]	40	87.79%	10.11%	5.06%	3.79%
Indian - Saxena et al., 1988 [11]	72	95.30%	3.46%	1.38%	11.79%
Japanese - Matsumura, 1991 [11]	614	79.10%	2.60%	17.70%	0.60%
Manjunath et al., 1993 [12]	172	93.55%	3.52%	2.93%	17.30%
Asala et al., 1996 [13]	212	82.10%	23.60%	-	5.70%
Korean - Lee et al., 2001 [14]	149	76.50%	-	-	40.30%
Turkish - Ersoy, 2003 [15]	300	87.35%	3.47%	8.98%	0.20%
Turkish males - Oguz, 2004 [16]	26	88%	10%	2%	0%
Saxena RC et al., 2003 [17]	203	84.72%	10.01%	5.17%	-
Kenyans - Mwachaka PM, 2009 [18]	79	66%	15%	12%	7%
Ankur Zalawadia et al., 2010 [19]	42	91.70%	2.40%	1.20%	4.80%
Hussain Saheb S et al., 2011 [20]	125	69.25%,	17.35%	9.70%	3.70%
Thai - W Apinhasmit et al., 2011 [21]	268	81.20%	1.10%	0.40%	17.40%
Mary Antony Praba et al., 2012 [22]	50	74%	3%	9%	14%
Sunday A Adejuwon et al., 2013 [23]	37	86.10%	8.30%	5.60%	-
Prashant Natekar et al., 2016 [24]	150	85.33%	8%	10.60%	51.54%
Turkey-Sindel et al., 2016 [25]	150	63%	2%	19%	16%
Present Study	80	72.50%	3.80%	-	23.80%

The presence of these variations may arise from a combination of genetic or environmental factors. Discrepancies in observed distances across different studies might be attributed to genetic influences, nutritional factors, geographic variations, and environmental conditions [26].

The chi-square test, exploring the correlation between the pterion type and side, yielded a non-statistically significant result (P=0.669), suggesting that the type of pterion is independent of the side of the head. This finding is consistent with the findings reported by Apinhasmit et al. in 2011, who also found no statistically significant correlation between the pterion type and side. Similarly, Murphy's analysis of the pterion in Australian aborigines found no evidence of a side effect on the prevalence of pterion type [21].

In our current investigation, the average distance between the pterion and the midpoint of the zygomatic arch was 37.33±4.8 mm when considering both sides. On the right side, the average distance was 37.69±5.01 mm, and on the left side, it was 36.96±4.8 mm. The posterior aspect of the frontozygomatic suture averaged 29.56±5.9 mm across both sides, with 20.42±5.8 mm on the right and 28.69±5.9 mm on the left. According to Sindel et al. 2016, the sphenoparietal type of pterion accounted for 63%, the frontotemporal type for 2%, the stellate type for 19%, and the epipteric type for 16% in our study [25]. Based on measurements, the pterion was located approximately 3.98 cm above the arcus zygomaticus and 3.4 cm behind the frontozygomatic suture. These findings differ from those of Eboh DE and Obaroefe M, who reported the pterion to be situated at 39.87±3.16 mm above the midpoint of the zygomatic arch and 31.56±2.47 mm above the posterior aspect of the frontozygomatic suture in skulls of the Nigerian population [27]. They are also lower than the measurements of Dutt V et al., who found the pterion in Indian-origin skulls to be 38.15±3.67 mm higher on the right side and 36.69±3.64 mm on the left [28].

In our study, the mean distance of the pterion from the external acoustic meatus was 52.65±3.5 mm when considering both sides, with 52.82±3.2 mm on the right side and 52.48±3.8 mm on the left side of the skull. The mean distance of the pterion from the nasion was 75.29±5.6 mm when considering both sides, with 76.04±5.6 mm on the right side and 74.54±5.6 mm on the left side. We found type 2 asterion to be the most common (82.1%), while type 1 was the least common (11.7%), consistent with previous studies incorporated in Table 6.

**Table 6:** Morphology of the asterion in various populations

Asterion			
Study	n (Sample size)	Type 1	Type 2
Kenyans - Mwachaka – 2009 [18]	79	20%	80%
Indians - Hussain Saheb S - 2010 [20]	125	23.15%	76.85%
Indian – Vivaan Dutta - 2017 [28]	78	13.46%	86.54%
North Americans - Berry – 1967 [29]	50	12%	88%
South Americans - Berry – 1967 [29]	53	7.50%	92.50%
Egyptians - Berry – 1967 [29]	250	14.40%	85.60%
Indians – Burma- Berry – 1967 [29]	51	14.70%	85.30%
Indians – Punjab- Berry – 1967 [29]	53	16.90%	83.10%
Iranian – Gharehdaghi 2020 [30]	210	14.7%	86.3%
Turks - Gumusburun – 1997 [31]	302	9.92%	90.08%
Mexican - Galindo-de León S – 2013 [32]	176	25.6%	74.4%

The hypothesis posits that the emergence of sutural bones at the asterion may be attributed to ongoing genetic and physiological processes, although the presence of pathological factors, such as hydrocephalus, could also be influential [28].

In our investigation, the mean distance between the asterion and the apex of the mastoid was 45.85±5.8 mm when considering both sides. On the right side, the average distance was 46.41±5.86 mm, and on the left side, it was 45.30±5.9 mm. The mean distance of the asterion from the posterior end of the zygoma was 56.31±5.9 mm for both sides combined, with 55.72±6.1 mm on the right side and 56.90±5.9 mm on the left side. Similarly, the mean distance between the asterion and the external occipital protuberance was 61.08±5.6 mm when considering both sides together, with 60.01±5.2 mm on the right side and 62.15±5.9 mm on the left side. Additionally, the mean distance of the asterion from the lambda was 78.37±4.7 mm for both sides combined, with 77.71±5.5 mm on the right side and 79.03±3.7 mm on the left side. These findings align with the study conducted by Galindo-de León S et al., 2013 at Universidad Autónoma de Nuevo León, Mexico, which reported a mean distance of the asterion from the apex of the mastoid at 51.53±4.97 mm from the root of the zygomatic arch at 54.74±4.46 mm, and from the external occipital protuberance at 61.51±7.44 mm [32].

In contrast, our results indicate greater distances compared to the study by Akkaşoğlu S et al. in 2019, which reported the distance from the asterion to the mastoid process as 43.65±6.75 mm on the left side and 45.01±6.04 mm on the right side. The distance from the asterion to the posterior end of the zygomatic arch was 43.97±7.37 mm on the left side and 43.95±7.02 mm on the right side. Their measurements for the distance between the asterion and the external occipital protuberance were 62.59±8.83 mm on the left side and 54.75±5.57 mm on the right side. Furthermore, the distance from the asterion to lambda was reported as 81.40±7.36 mm on the left side and 82±4.96 mm on the right side in their study [8].

For neurosurgeons, knowing the precise location of these structures and their relationship to bony landmarks used in surgical approaches is extremely valuable. Some authors have sought and established these topographical landmarks to locate different portions of the venous sinus [32]. However, a limitation of our study is that the effectiveness of the results could have been enhanced with a larger number of skulls included.

## Conclusions

This study affirms that the morphological and morphometric characteristics of the pterion and asterion in Indian skulls closely resemble those observed in other populations, consistent with findings from previous studies across diverse populations. In Indian skulls, the predominant pterion type is sphenoparietal, while the most common asterion type is type 2, distinguished by the absence of a sutural bone at the asterion. The association test results examining the relationship between pterion type and side, as indicated by the chi-square value, were not statistically significant (p>0.05).

In summary, the pterion and asterion serve as crucial anatomical landmarks on the human skull, carrying significant clinical implications across various medical fields, particularly in neurosurgery and radiology. The pterion, being associated with the middle meningeal artery, is susceptible to fractures resulting from head trauma, potentially leading to life-threatening epidural hematomas. Additionally, neurosurgeons utilize the pterion as an entry point for accessing specific brain regions. Similarly, the asterion plays a vital role in neurosurgical procedures involving the posterior fossa and cerebellum, aiding in surgical planning and execution. Understanding the clinical significance of these landmarks is crucial for healthcare professionals, ensuring accurate diagnosis, safe surgical procedures, and effective management of head trauma patients through a comprehensive approach integrating radiological assessment.
